# Formaldehyde Connection: Modeled Exposure Linked to Lower Respiratory Infections in Infants

**DOI:** 10.1289/ehp.119-a489a

**Published:** 2011-11-01

**Authors:** Julia R. Barrett

**Affiliations:** Julia R. Barrett, MS, ELS, a Madison, WI–based science writer and editor, has written for *EHP* since 1996. She is a member of the National Association of Science Writers and the Board of Editors in the Life Sciences.

Lower respiratory infections (LRIs) are common among infants, with risk increased by factors such as daycare attendance, older siblings, and parental history of asthma. The incidence of infections can also be exacerbated by environmental pollutants such as tobacco smoke, nitrogen dioxide, and ozone. Formaldehyde, a known respiratory tract irritant, is ubiquitous in indoor environments, but little is known about the effects of chronic exposure in infants. A new study reveals that such exposure is associated with more LRIs during infancy [*EHP* 119(11):1653–1658; Roda et al.].

The current study used data for 2,940 infants enrolled in Pollution and Asthma Risk: An Infant Study (PARIS), a cohort of healthy, full-term babies born at five Parisian hospitals from 2003 to 2006. Parental history of allergic conditions was obtained by interview, while medical records provided additional data on the newborns and their mothers. Multiple mailed questionnaires were used to gather information from parents about recent respiratory infection, wheezing, and eczema in their children at ages 1, 3, 6, 9, and 12 months. Details about home characteristics and family living conditions were collected by phone interview when infants were 1 month old, and mailed questionnaires captured changes at 3, 6, 9, and 12 months. Aldehyde air sampling measurements were conducted at 1, 6, 9, and 12 months in the homes of a subset of randomly selected infants, and data for 174 homes were joined with interview and questionnaire information to construct formaldehyde exposure models for all cohort infants.

The median value of formaldehyde measured in the subset of homes was 19.5 µg/m^3^, with an interquartile range of 14.4–26.8 µg/m^3^. Overall, more than 45% of the infants experienced at least one LRI, and nearly half of those infections included wheezing. After known risk factors were considered, LRI and LRI with wheezing increased by 32% and 41%, respectively, for each interquartile increase in estimated formaldehyde levels.

Although the models used to predict formaldehyde levels for most of the homes demonstrated adequate performance, they were based on only a few hundred actual measurements. Thus, the associations between formaldehyde and LRIs are based upon statistical estimates of exposure rather than on actual measurements. However, the model performed well for established LRI factors, suggesting that the findings for formaldehyde exposure were likewise valid.

